# Comparison of resting-state EEG between adults with Down syndrome and typically developing controls

**DOI:** 10.1186/s11689-021-09392-z

**Published:** 2021-10-14

**Authors:** Sarah Hamburg, Daniel Bush, Andre Strydom, Carla M. Startin

**Affiliations:** 1 The London Down Syndrome Consortium (LonDownS), London, UK; 2 Institute of Cognitive Neuroscience, University College London, London, UK; 3 Queen Square Institute of Neurology, University College London, London, UK; 4 Department of Forensic and Neurodevelopmental Sciences, Institute of Psychiatry, Psychology and Neuroscience, King’s College London, London, SE5 8AF UK; 5 Department of Psychology, University of Roehampton, London, UK

**Keywords:** Down syndrome, Trisomy 21, EEG, Resting state, Alpha peak

## Abstract

**Background:**

Down syndrome (DS) is the most common genetic cause of intellectual disability (ID) worldwide. Understanding electrophysiological characteristics associated with DS provides potential mechanistic insights into ID, helping inform biomarkers and targets for intervention. Currently, electrophysiological characteristics associated with DS remain unclear due to methodological differences between studies and inadequate controls for cognitive decline as a potential cofounder.

**Methods:**

Eyes-closed resting-state EEG measures (specifically delta, theta, alpha, and beta absolute and relative powers, and alpha peak amplitude, frequency and frequency variance) in occipital and frontal regions were compared between adults with DS (with no diagnosis of dementia or evidence of cognitive decline) and typically developing (TD) matched controls (*n* = 25 per group).

**Results:**

We report an overall ‘slower’ EEG spectrum, characterised by higher delta and theta power, and lower alpha and beta power, for both regions in people with DS. Alpha activity in particular showed strong group differences, including lower power, lower peak amplitude and greater peak frequency variance in people with DS.

**Conclusions:**

Such EEG ‘slowing’ has previously been associated with cognitive decline in both DS and TD populations. These findings indicate the potential existence of a universal EEG signature of cognitive impairment, regardless of origin (neurodevelopmental or neurodegenerative), warranting further exploration.

**Supplementary Information:**

The online version contains supplementary material available at 10.1186/s11689-021-09392-z.

## Introduction

Down syndrome (DS) is caused by an extra copy of chromosome 21 and is the most common genetic cause of intellectual disability (ID) worldwide, affecting 1 in 800 births [1]. Due to a ‘triple dose’ of genes on this chromosome, almost all individuals with DS have an ID (clinically defined as an IQ less than 70 and impairments in everyday adaptive abilities), in addition to an ultra-high risk of developing Alzheimer’s disease (AD) with a lifetime prevalence of 90% [2]. Understanding brain function in people with DS is important for elucidating these characteristics, and electrophysiological measures in particular allow us to examine potential mechanisms related to function. Understanding electrophysiological characteristics associated with DS may therefore provide mechanistic insights into both cognitive ability and decline.

Resting-state electroencephalography (EEG) paradigms provide a general measure of brain activity (i.e. activity that is not associated with any particular sensory modality). As resting-state paradigms are passive, they are inherently free from the need for participants to understand and retain task instructions (e.g. pressing a button in response to a target), thus reducing any confounding influences of individual differences in ID level and motor skills [3]. Such paradigms are therefore suitable for use with the majority of individuals with DS.

EEG recordings reveal oscillatory brain activity. The organisation of this activity represents the means by which neuronal networks dynamically communicate and interact [4]. For example, networks of inhibitory interneurons generate rhythmic inhibitory post-synaptic potentials (IPSPs). These rhythmic IPSPs provide windows of alternating reduced and enhanced excitability, offering a temporal framework for the ‘chunking’ of neuronal activity. This ‘chunking’ enables the effective communication of local information to distributed regions. It is posited that these mechanisms enable the brain to integrate a large number of distributed local processes into global states [4]. Such brain rhythms are characterised by distinct frequency bands, associated with differing underlying mechanisms of generation and brain functions; typically, delta (< 4 Hz), theta (4-8 Hz), alpha (8-13 Hz), beta (13-30 Hz) and gamma (> 30 Hz). It is of note, however, that brain rhythms may operate across a wider frequency range than these discrete categories, with substantial inter-subject variability [5].

Using EEG to identify individual and group differences in oscillatory brain activity has been of interest to researchers since EEG was invented in the 1920s. Power in both slow (delta and theta) and fast (beta and gamma) EEG bands has generally been reported as greater in participants with DS compared to typically developing (TD) controls [6–14]. This pattern is well established for slower frequencies, though inconsistencies in the literature exist for faster frequencies, with Babiloni et al. [12, 13] reporting less power in beta and gamma bands in individuals with DS compared to TD controls.

As alpha waves can be visually identified in EEG recordings, they have been of particular interest to DS researchers from the earliest EEG studies [15]. Alpha activity is commonly associated with both IQ and memory performance in the TD population [16–18], in addition to between people with DS [19]. Differences in alpha activity between people with DS and TD controls are commonly reported, though specific findings are inconsistent. Whilst the majority of studies have reported less alpha power in DS [8, 11, 12], others have found significantly more alpha power in DS [9] or no difference between DS and TD controls [10]. For alpha peak frequency (the frequency at which peak amplitude occurs within this band), many studies have found people with DS have a significantly slower peak frequency [6, 7, 11, 14, 20], though others reported no significant difference [10, 13].

These inconsistencies in findings, for alpha power in particular, are in part likely due to methodological differences between EEG studies. Differences in study characteristics are common, including frequency band classification, power measure (i.e. relative or absolute), scalp regions examined, participant age and whether any participants show signs of cognitive decline. For example, Politoff et al. [10] reported no significant differences between people with DS and TD controls for relative power (where power is calculated relative to the total EEG power for each participant) but found differences for absolute power. There is therefore value in examining both type of power measure. Previous research also shows topographical differences are important to consider—differences in alpha activity between people with DS and TD controls appear most apparent in posterior regions [8, 11] and may differ between occipital and parietal electrode derivations [6], whilst delta differences may be most apparent in frontal and centro-anterior regions [8, 11, 12], theta differences in centro-posterior regions [8, 11], and beta differences in parieto-temporal regions [8, 11].

Given the previous discrepancies in the literature regarding differences in EEG measures between people with DS and TD controls, and the potential contribution of methodological variations to these discrepancies, it is important to conduct research accounting for these variations to understand differences in EEG activity between people with DS and TD controls. We therefore compared EEG activity between adults with DS and TD controls using commonly used frequency band classifications, and both absolute and relative power, in addition to including two scalp regions (occipital and frontal). To reduce a potential confounding effect of cognitive decline, we used a sample of adults with DS with no noticeable cognitive decline. Understanding these differences in activity between adults with DS and TD controls will not only provide mechanistic insights into cognitive ability but also help elucidate the significance of studies examining individual differences between people with DS. In turn, this may help inform biomarker and drug target research.

Based on previous findings, it was hypothesised that individuals with DS would have less alpha power (8-13 Hz) but more power in delta (0.5-4 Hz), theta (4-8 Hz) and beta (13-30 Hz) bands compared to TD controls. Results were not expected to significantly differ between absolute and relative power measures, or between occipital and frontal electrode montages. Gamma activity was not investigated as it shares a similar frequency to muscle artefacts, which are common in electrophysiological recordings in people with DS due to lower compliance with the instruction to remain still.

## Methods

### Participants

Participants with DS were recruited from an existing pool of UK adults with DS who had participated in an initial cognitive assessment [3]. All participants had genetically confirmed trisomy 21 and were aged 16 and over. Participants with an acute physical or mental health condition were excluded, as were participants with a clinical diagnosis of dementia or the presence of cognitive decline associated with dementia. The presence of cognitive decline was determined by the Cambridge Examination of Mental Disorders of Older People with Down Syndrome and Others with Intellectual Disabilities (CAMDEX-DS [21]), which is considered a valid and reliable tool for assessing cognitive decline in adults with DS [21]. It is an informant-based questionnaire which enquires about decline (with respect to an individuals’ best level of functioning) within the following domains: everyday skills, memory, orientation, general mental functioning, language, perception, praxis, executive functions, personality, behaviour and self-care. Any change in any one of these domains was scored as presence of decline. All participants were required to show no decline on this questionnaire to be included in the study. The resting-state EEG recordings from all participants with DS selected for this study has also previously been used in a separate investigation into differences between individuals with DS [19].

TD control group participants were selected from the Multimodal Resource for Studying Information Processing in the Developing Brain (MIPDB) [22]. The MIPDB is a large open source dataset provided by the Child Mind Institute. This dataset aims to advance the study of clinical cognitive neuroscience, and contains high-density task-based and task-free raw EEG data collected from TD individuals aged 6-44 years.

All participants were required to have sufficient EEG data (at least 24 s of artefact-free data) and for no measured EEG variables to fall > 3 SD from the group mean (indicative of outlier activity). All participants meeting inclusion criteria were considered for matching. In total, 25 individuals from each pool were chronologically age-matched to within 1 year, and sex-matched at a sub-group level split by age (16-25 years, 26-35 years, 36 years and over).

### EEG acquisition and pre-processing procedure

Data from both groups was acquired using 128-channel EEG Geodesic Hydrocel nets (Electrical Geodesics, Inc., Eugene, OR, USA) with an appropriate size selected by measuring head circumference. In both datasets, electrode impedances were maintained below 50 kΩ during recording; the EEG signal was referenced to the vertex, and was recorded with a bandpass filter of 0.1 to 100 Hz. An amplifier gain of 10,000 was used for both datasets. Data from adults with DS was sampled at a rate of 250 Hz, whilst TD control data was sampled at a rate of 500 Hz.

During the resting-state task, participants of both groups repeated multiple eyes closed (EC) recording blocks. However, the first 11 participants with DS had one continuous 5.5 min EC block, which was then changed to multiple shorter blocks due to poor compliance and drowsiness (see Hamburg et al. [19] for details). All preprocessing was performed using EEGLAB [23] for MATLAB (MathWorks, Natick, MA) and was identical for both groups (see Hamburg et al. [19]). Briefly, the continuous EEG signal was digitally filtered using a low-pass filter of 30 Hz. All data from six channels situated around the ears were removed due to poor fit (as a result of morphological differences in people with DS). Movement and blink artefacts were removed manually based on visual inspection. Bad channels were also identified based on visual inspection and were replaced using spherical spline interpolation. Remaining channels were re-referenced to the average electrode excluding VEOG and HEOG channels.

### EEG analysis

**Fig. 1:**
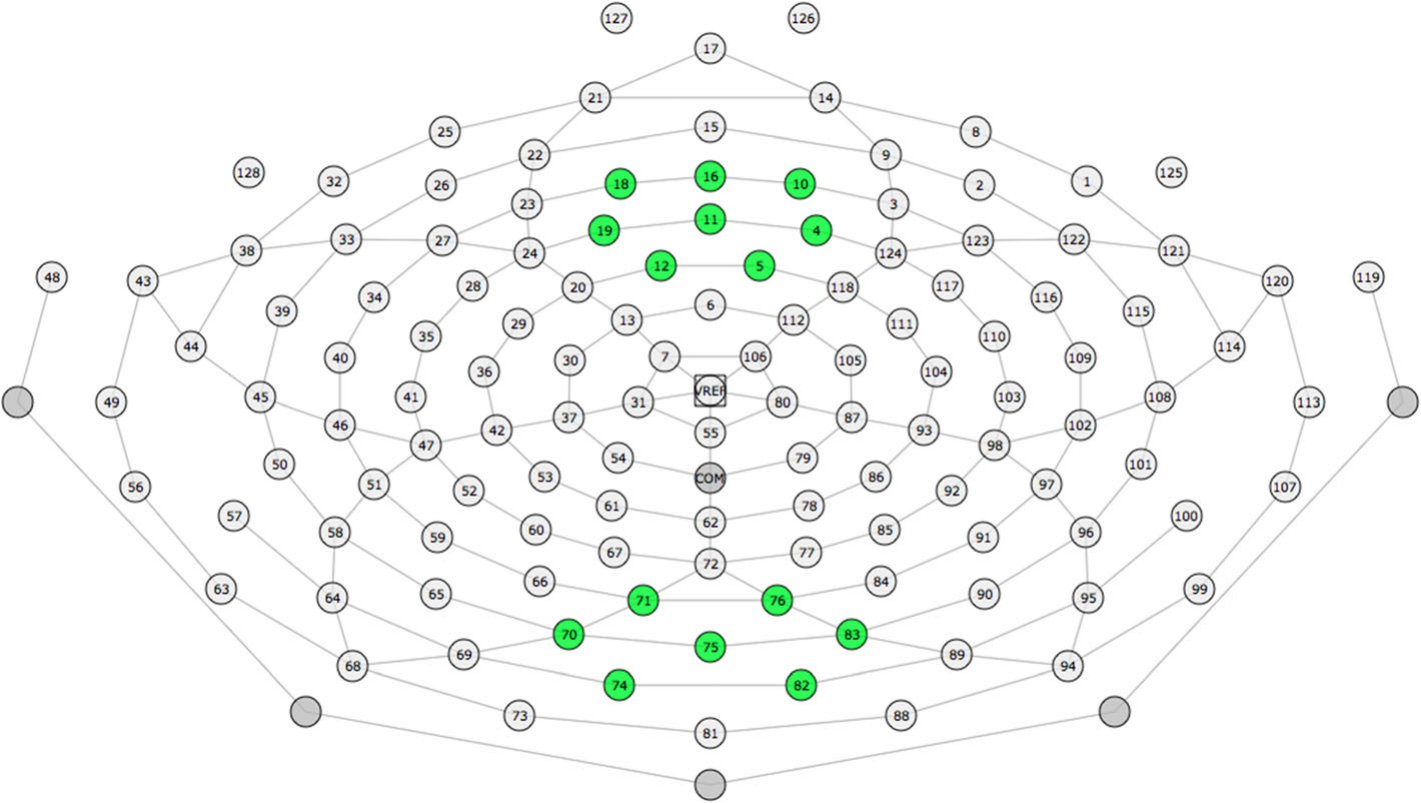
Electrode map illustrating occipital and frontal montages. Electrode map illustrating individual cap electrodes for occipital (bottom cluster; 70 (O1), 71, 74, 75 (Oz), 76, 82, 83 (O2)) and frontal (top cluster; 4 (F2), 5, 10, 11 (Fz), 12, 16 (AFz), 18, 19 (F1)) montage averages used within this analysis. Corresponding 10-20 system shown in brackets where applicable

Analysis was carried out using MATLAB (MathWorks, Natick, MA). For each individual, absolute and relative power measures for each frequency band of interest were obtained (delta 0.5-4 Hz; theta 4-8 Hz; alpha 8-13 Hz; beta 13-30 Hz) for each region (frontal and occipital; see Fig. 1 for electrode montages). Additionally, alpha peak features were calculated; this was defined as the frequency (Hz) of the peak amplitude within the 8-13 Hz range.

Specifically, absolute power measures were obtained by convolving the raw signal from artefact free, non-overlapping 2 s epochs for each channel with a five cycle Morlet wavelet. Power spectra were then averaged across all 2 s epochs, yielding a single average power spectrum for every electrode for each individual. Relative power measures were obtained for every electrode for each individual by dividing absolute power values by the total absolute power across the 0.1-30 Hz frequency range.

Some participants with DS did not have a measurable alpha peak. Standard methods would assign peak frequency to the lower boundary (i.e. 8 Hz) for these individuals, as brain signals show a decrease in power with increasing frequency. Alpha peak features were therefore obtained for all individuals by removing the linear trend from individual power spectra to achieve ‘spectral normalisation’ [24]. This method allowed an accurate representation of these values to be obtained for all individuals, including those whose peak characteristics were initially lost within the natural EEG background.

### Statistics and visualisation

Customised MATLAB (MathWorks, Natick, MA) scripts were used to produce power-frequency spectrum plots. All statistical analyses were performed with SPSS.

In order to determine whether there was any effect of EC paradigm on EEG variables within individuals with DS (i.e. one segment of 5.5 minutes compared to 11 segments of 30 seconds), independent sample *t* tests were used to compare absolute and relative power values between DS participants completing a full-block (*n* = 11) and those completing a split-block (*n* = 14) paradigm. As one *t* test was significant (higher relative frontal theta power was found for the full-block paradigm (*M* = 0.26 (0.02 SD)) compared to the split-block paradigm (*M* = 0.24 (0.01 SD)), (*t* (14.96) = 2.35, *p* = .033 (95% CI < 0.01, 0.02)), EC paradigm was added as a covariate for all comparisons when activity was compared between groups.

All TD control participants were assigned to the split-block protocol. ANCOVAs were used to statistically compare differences between groups. This was performed for each EEG variable at each region (occipital and frontal), using both absolute and relative power values and alpha peak amplitude, and alpha peak frequency values. Where the covariate (EC paradigm) was significant, this was left in the model, and where this was not significant, this was removed from the model. Partial eta squared values for each variable were used to provide an indication of effect size.

## Results

### Preliminary analysis

Final analyses were carried out on 25 individuals from each group. Table 1 shows the demographics of all participants included in the final analysis.

**Table 1:** Participant demographics for each group

Group	***n***	Mean age (SD)	Age range	Sex
DS	25	27.76 (8.45)	17-44	12 M; 13 F
Control	25	27.68 (8.34)	16-44	14 M; 11 F

Participants were matched individually for age (years; within 1 year) and on a sub-group level for sex (sub-groups were age 16-25 years, 26-35 years, 36 years and over)

According to carer report of participants with DS, level of ID was mild (*n* = 13), moderate (*n* = 10), and severe (*n* = 2).

### EEG measures

**Fig. 2:**
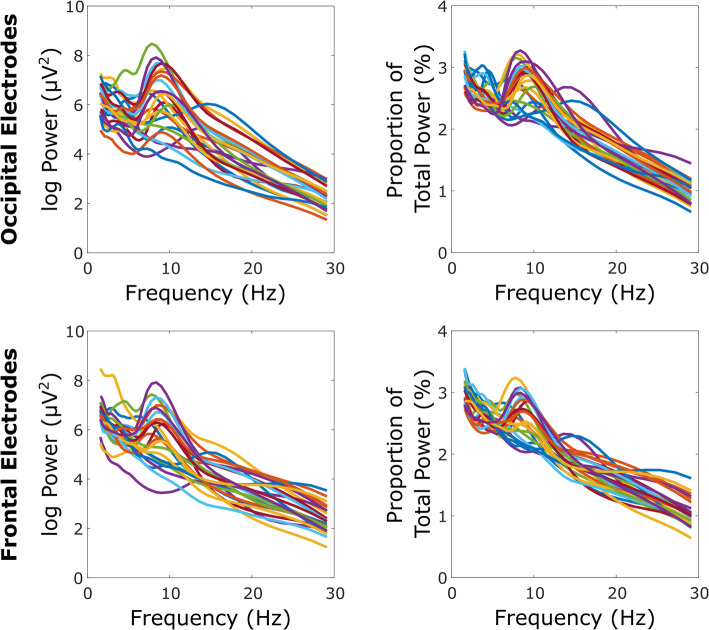
DS power-frequency spectra. DS group power-frequency spectra for occipital (top) and frontal (bottom) regions. Absolute (left) and relative (right) values are shown for each individual

**Fig. 3:**
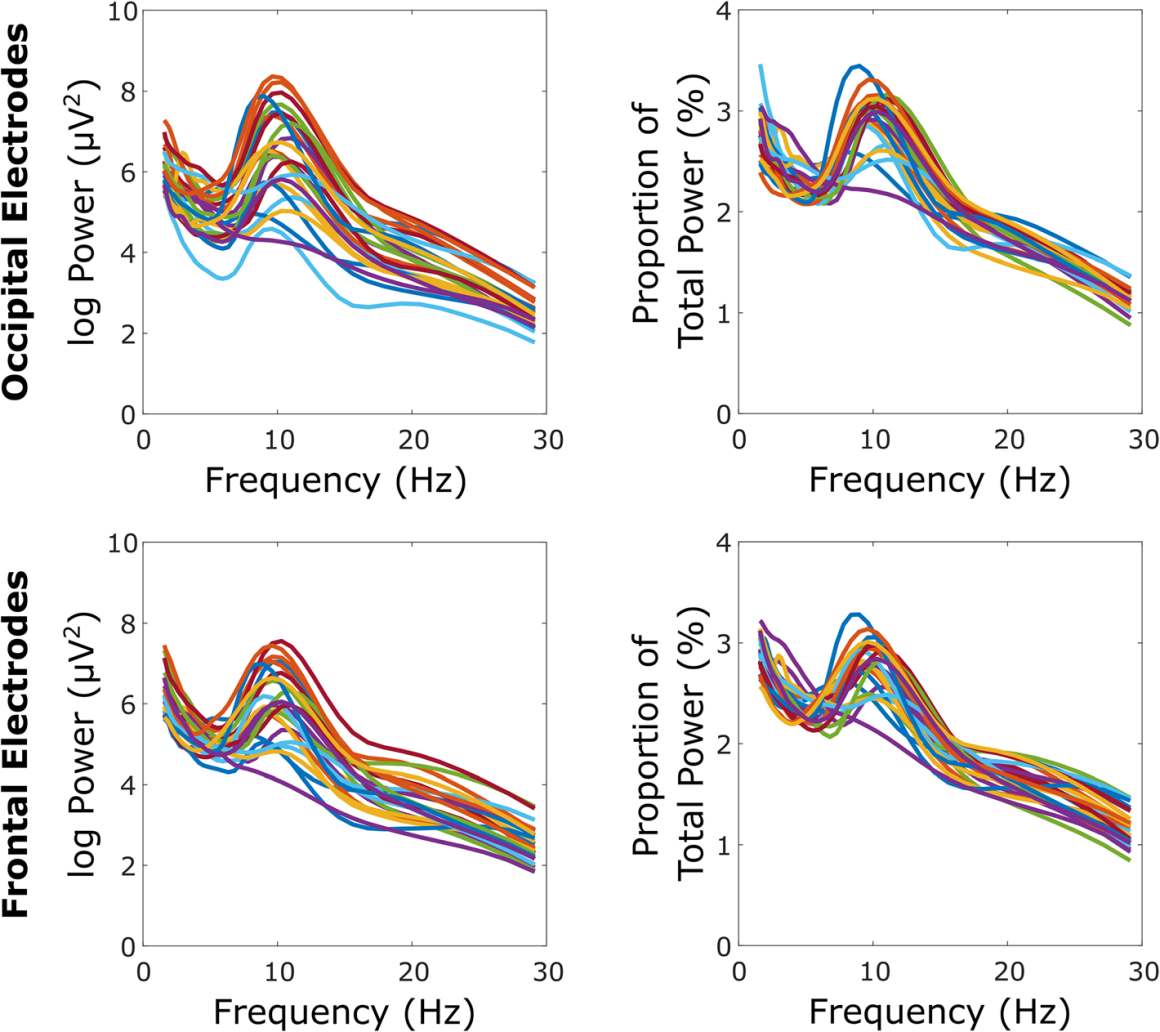
TD power-frequency spectra. TD control group power-frequency spectra for occipital (top) and frontal (bottom) regions. Absolute (left) and relative (right) values are shown for each individual

Table 2 shows absolute and relative values for each EEG measure by region within each group, in addition to statistical analysis of EEG variables by region. Standard deviations in the DS group appeared to be higher than those in the control group, particularly for peak frequency, indicative of more variability. Figure 2 (DS group) and Fig. 3 (TD control group) further illustrate increased variability within the DS group, apparent from individual power spectra. As a consequence, further analysis was undertaken to compare alpha peak frequency variance between groups, with highly significant between group effects found for both occipital (*F* (24, 24) = 59.98, *p* < .001) and frontal (*F* (24, 24) = 29.15, *p* < .001) regions.

**Table 2:** EEG values for each group

EEG measure (***n*** = 25 per group)	DS mean (SD)		TD control mean (SD)		Occipital		Frontal	
	Occipital	Frontal	Occipital	Frontal	Equation	Partial eta squared	Equation	Partial eta squared
Absolute delta power (0.5-4 Hz range; log μV^2^)	5.71 (0.52)	6.23 (0.59)	5.41 (0.36)	5.75 (0.39)	*F* (1, 47) = 1.91, *p* = .037*	.039 group	*F* (1, 47) = 11.14, *p* = .002**	.192 group
Relative delta power (0.5-4 Hz range; log μV^2^)	0.39 (0.02)	0.39 (0.02)	0.37 (0.02)	0.37 (0.02)	*F* (1, 47) = 8.18, *p* = .006**	.148 group	F (1, 47) = 8.34, *p* = .006**	.151 group
Absolute theta power (4-8 Hz range; log μV^2^)	5.29 (0.71)	5.69 (0.65)	4.89 (0.44)	5.15 (0.34)	^1^Group: *F* (1, 47) = 0.94, *p* = .338 Paradigm: *F* (1, 47) = 4.73, *p* = .035*	.020 group; .091 paradigm	F(1, 47) = 6.78, *p* = .012*	.126 group
Relative theta power (4-8 Hz range; log μV^2^)	0.25 (0.01)	0.26 (0.01)	0.24 (0.01)	0.24 (0.01)	^1^Group: *F* (1, 47) = 13.98, *p* = .001*** Paradigm: *F* (1, 47) = 6.77, *p* = .012*	.229 group; .126 paradigm	^1^Group: *F* (1, 47) = 9.25, *p* = .004** Paradigm: *F* (1, 47) = 7.83, *p* = .007**	.164 group; .143 paradigm
Absolute alpha power (8-13 Hz range; log μV^2^)	5.19 (0.91)	5.43 (0.96)	5.76 (0.88)	5.82 (0.85)	*F* (1, 47) = 5.20, *p* = .027*	.100 group	*F* (1, 47) = 1.87, *p* = .179	.038 group
Relative alpha power (8-13 Hz range; log μV^2^)	0.15 (0.02)	0.15 (0.02)	0.19 (0.02)	0.16 (0.01)	*F* (1, 47) = 61.04, *p* ≤ .001***	.565 group	*F* (1, 47) = 12.45, *p* ≤ .001***	.209 group
Absolute beta power (13-30 Hz range; log μV^2^)	3.27 (0.52)	3.46 (0.54)	3.56 (0.45)	3.64 (0.46)	*F* (1, 47) = 4.03, *p* = .050*	.079 group	*F* (1, 47) = 0.33, *p* = .569	.007 group
Relative beta power (13-30 Hz range; log μV^2^)	0.19 (0.02)	0.19 (0.02)	0.20 (0.01)	0.20 (0.01)	*F* (1, 47) = 8.32, *p* = .006**	.150 group	*F* (1, 47) = 4.45, *p* = .040*	.087 group
Absolute alpha peak amplitude (8-13 Hz range; log μV^2^)	0.11 (0.06)	0.12 (0.06)	0.24 (0.08)	0.23 (0.09)	*F* (1, 47) = 27.88, *p* ≤ .001***	.372 group	*F* (1, 47) = 19.05, *p* ≤ .001***	.288 group
Relative alpha peak amplitude (8-13 Hz range; log μV^2^)	0.03 (0.00)	0.03 (0.00)	0.03 (0.00)	0.03 (0.00)	*F* (1, 47) = 9.72, *p* = .003**	.171 group	*F* (1, 47) = 8.98, *p* = .004**	.160 group
Alpha peak frequency (8-13 Hz range; Hz)	10.32 (1.07)	10.44 (1.11)	10.27 (0.14)	10.30 (0.21)	^1^Group: *F* (1, 47) = 1.52, *p* = .224 Paradigm: *F* (1, 47) = 7.38, *p* = .009**	.031 group; .136 paradigm	*F* (1, 47) = 0.191, *p* = .664	.004 group

Mean (SD) values for all EEG variables investigated, shown for each region (occipital and frontal) and group (DS and TD control). Occipital and frontal region group comparison: Results of ANCOVAs between groups for each EEG variable for occipital region and frontal regions shown. ^1^Covariate significant so kept in model. Asterisk used to denote significance level (≤.05*, ≤.01**, ≤.001***). Effect sizes illustrated with partial eta squared value

**Fig. 4:**
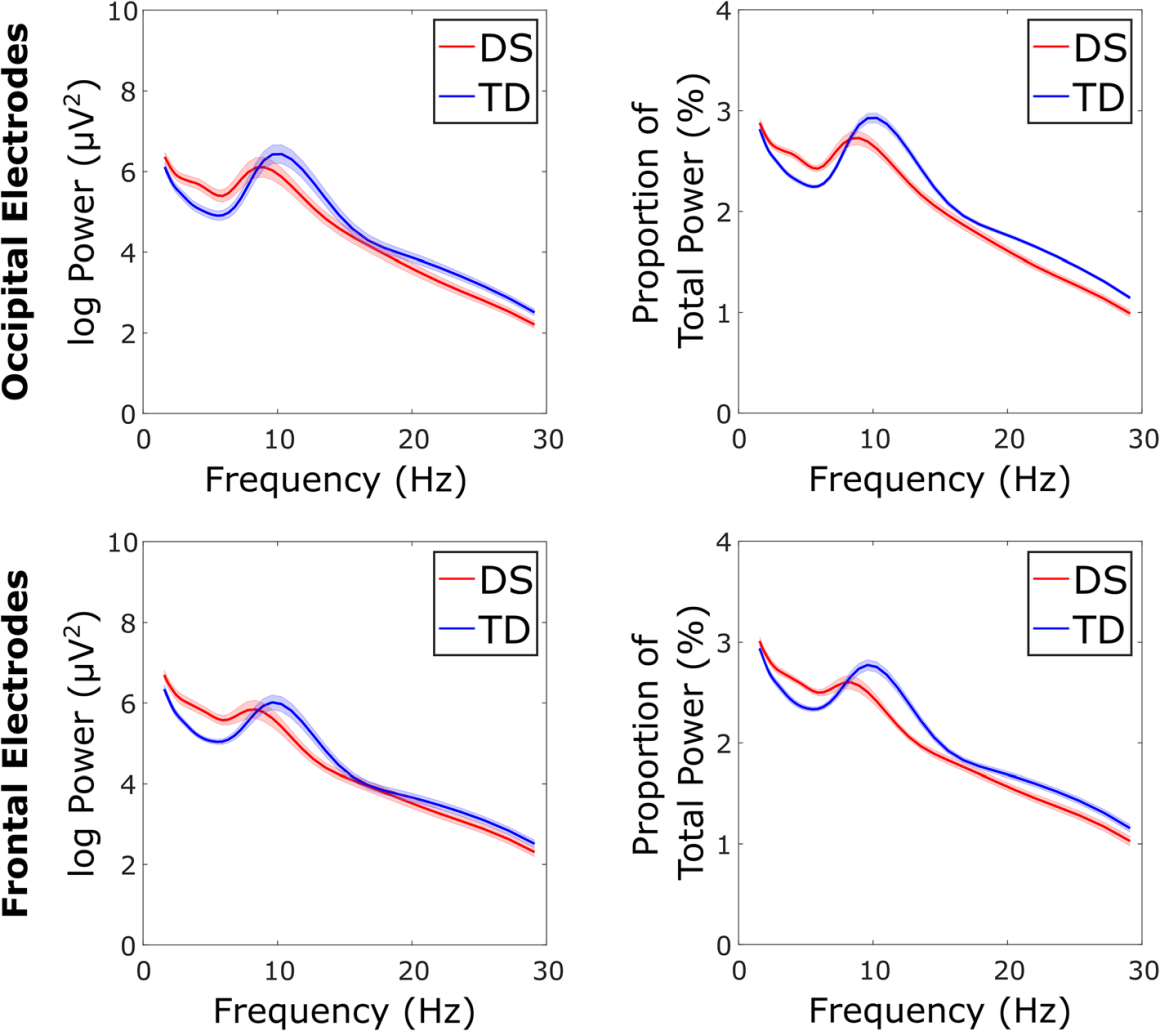
Group comparison of power-frequency spectra. Comparison between DS group (red) and TD control group (blue) grand average power-frequency spectra for occipital (top) and frontal (bottom) regions. Absolute (left) and relative (right) spectra are both shown

The overall group differences in the power-frequency spectra between DS and TD control participants are illustrated by Fig. 4.

Statistical analysis of EEG variables from the occipital region revealed significantly higher absolute and relative delta power, and relative theta power, and significantly lower absolute and relative power in alpha and beta bands, for those with DS compared to TD controls (see Fig. 4 and Table 2). Those with DS also showed a significantly lower alpha peak amplitude. The effect sizes were greatest for relative alpha power, with group accounting for 56.5% of variance.

Results for the frontal region followed the same pattern (see Tables 2 and 3). All group differences (including both absolute and relative values) were statistically significant, apart from absolute alpha and beta power and alpha peak frequency. Overall, in this region, absolute and relative delta and theta power values were significantly higher in individuals with DS, whereas relative alpha and beta power values, and both absolute and relative alpha peak amplitude, were all significantly lower. The effect sizes were greatest for absolute alpha peak amplitude and relative alpha power, with group accounting for 28.8% and 20.9% of variance in these variables respectively.

**Table 3:**
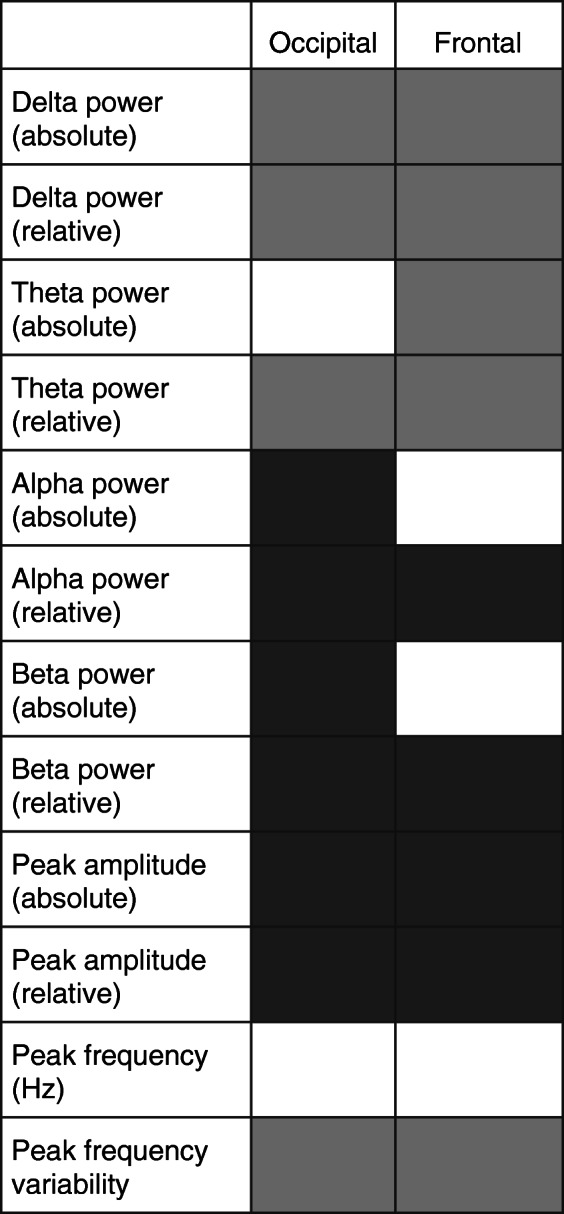
Summary of significant results

Light grey shows EEG measures that were significantly higher and dark grey shows measures that were significantly lower in participants with DS compared to TD controls

It is worth noting that paradigm had a significant effect on occipital theta power, with both absolute and relative theta values higher for the full-block compared to the split-block paradigm for participants with DS (absolute values 5.57 log μV^2^ (0.63 SD) full-block and 5.08 log μV^2^ (0.71 SD) split-block; relative values 0.26 log μV^2^ (0.02 SD) full-block and 0.25 log μV^2^ (0.01 SD) split-block). Paradigm also had a significant effect on relative theta power in the frontal region, with higher values for the full-block compared to the split-block paradigm (0.26 log μV (0.02 SD) full-block; 0.24 log μV^2^ (0.01 SD) split-block). Additionally, there was a significant relationship between alpha peak frequency and paradigm in the occipital region—those with DS completing the full-block paradigm had a faster peak (10.76 Hz (1.27 SD)) compared with those completing the split-block paradigm (9.98 Hz (0.76 SD)). It is noteworthy that participants with DS completing the full-block also had higher standard deviation of occipital peak frequency (1.27 vs. 0.76), indicating more variability in this particular measure.

## Discussion

This study aimed to characterise EEG differences between adults with genetically confirmed trisomy 21 with no evidence of dementia, and matched TD controls. We show an overall ‘slower’ EEG spectrum in both occipital and frontal regions (higher 0.5-8 Hz power and lower 8-30 Hz power) in people with DS. Alpha band activity in particular shows strong group differences, as shown by the greatest effect sizes for group. We illustrate the value of using high-density EEG recordings to examine topographical differences and of utilising relative power measures in this population.

Higher delta activity [8–14], higher theta activity [6–14], lower alpha activity [8, 11, 12], and lower beta activity [12, 13] have previously been reported in people with DS compared to TD controls. However, more evidence exists from previous research for higher than lower beta activity in people with DS [6–11, 14].

Interestingly, studies have linked ‘slower’ EEG spectra with cognitive impairment within the TD population—with increased delta and decreased alpha associated with poor memory performance [25], and increased delta and theta, and decreased alpha, associated with mild cognitive impairment (MCI) [26, 27]. Such differences may also become more pronounced with progression from MCI to AD in the TD population [28, 29]. It is therefore possible that cognitive impairment has similar EEG signatures whether due to ID or neurodegenerative disease, characterised by a ‘slower’ spectra with more activity at lower frequencies. Furthermore, additional ‘slowing’ of the EEG spectra has also been linked to dementia in people with DS [30].

According to effect sizes, EEG characteristics most strongly associated with group were those related to alpha activity (occipital and frontal relative alpha power, and frontal peak amplitude). As discussed previously, differences in alpha band activity between individuals with DS and TD controls are commonly reported. Alpha peak frequency was not significantly associated with group in either region, which is in line with two previous studies [10, 13], though other studies have reported a slower peak frequency in individuals with DS [6, 7, 11, 14, 20]. The difference in peak frequency variability (as measured using the SD) in individuals with DS compared to TD controls is large (1.07 Hz SD in DS; 0.14 Hz SD in TD controls), which may have impacted statistical power. Additionally, the highly significant group difference in alpha peak frequency variance suggests that peak frequency may be unstable in people with DS. This may be of particular importance as alpha peak frequency has been posited to act as an anchor around which the EEG spectrum is organised [17], and in turn the organisation of EEG activity represents the means by which neuronal networks dynamically communicate and interact [4].

As all individuals with DS are expected to have AD neuropathology (amyloid plaques and tau tangles) by age 35 [31], the extent to which the group differences reported here are related to ID associated with the presence of an extra chromosome 21 and/or subclinical AD-neuropathology, remains unclear. Potential ID-related mechanisms include delayed and reduced brain maturation (with maturation in TD children associated with age-related reductions in delta and theta power, and age-related increases in alpha and beta power [32]) and over-inhibition [33–35]. Studies involving alternative populations of individuals with ID (e.g. fragile X) would help elucidate whether these findings are unique to individuals with DS or are associated with ID in general. As the mean age of participants in this study is 27 years, which is prior to when significant amyloid-burden is expected in adults with DS [31], neuropathological mechanisms are unlikely. However, amyloid deposition can occur from childhood in DS [36] and therefore results may be confounded by this. Studies combining EEG with amyloid imaging (e.g. PET) could help explore this further. Future studies would also benefit from following individuals longitudinally, or examining different age groups cross-sectionally (including childhood and old age), to fully elucidate maturational and ageing influences.

For regional differences, stronger effect sizes were found for higher delta activity in adults with DS in frontal compared to occipital regions, which is in keeping with previous literature [8, 11, 12]. Frontally, differences in absolute values of alpha and beta power (although lower in DS) were not significant, yet reached significance occipitally, with greater effect sizes in occipital regions. Previous studies have also reported that differences in alpha and beta between people with DS and TD controls may be most apparent in posterior regions [8, 11]. It is of note that participants with DS had larger SD values in frontal regions compared to occipital regions, which may have impacted statistical power here.

Effect sizes were generally larger for relative power values (possibly due to normalisation of relative values reducing variability and consequently increasing statistical power). Due to the high degree of variability in EEG measures of participants with DS, utilising relative values may be particularly beneficial in this population when comparing to TD subjects. Relative power helps account for differences in broadband power across participants, therefore, helping to control for inter-individual variability in brain anatomy, which is particularly apparent in individuals with DS [37]. This anatomical variability may contribute to the higher EEG measure SD values found in this group. It is worth noting that absolute power values are likely to be of value when investigating individual differences between people with DS, as this variability is of interest.

The use of open source neuroimaging datasets is increasingly common and allows small exploratory studies of clinical population access to a large control group to obtain closely matched control subjects. Such datasets offer numerous benefits to researchers including increased efficiency, transparency and reproducibility [38]. Although variation in recording paradigm within the group with DS is a potential study limitation, effects were controlled for through the inclusion of this as a covariate. Furthermore, it appears likely that splitting the recording reduced participant drowsiness as intended (research suggests theta power is increased with light drowsiness [39] and theta power was higher for the full-block paradigm). Serendipitously, this provides useful information pertaining to the most appropriate design for resting-state studies in people with DS.

This study benefitted from only including individuals with genetically confirmed trisomy 21 and the exclusion of individuals with cognitive decline (as assessed using the CAMDEX-DS) or a diagnosis of dementia. This is important as cognitive decline in DS has been associated with changes in EEG activity [30]. This ensured results were not influenced by any individuals with a rarer form of DS (for example mosaicism), and results are valid for individuals with DS prior to dementia onset. These variables are not commonly controlled for within DS studies, despite them substantially impacting the validity of findings. An additional strength of this study is that peak frequency measures were obtained by removing the individual linear trend from the EEG spectrum to achieve ‘spectral normalisation’. This method has not been utilised in DS studies previously but is particularly useful in this population due to many individuals having a small peak that is not measurable beyond the natural background EEG noise.

A key limitation of this study is that there was no correction for multiple comparisons, due to the exploratory nature of this investigation—future studies should therefore prioritise replication of these findings. Future studies may also benefit from investigating differences in eyes-open EEG, and the examination of gamma activity in this population where possible. There is also an indication that the parietal region may be an area of particularly strong group differences (see Supplementary information), which future research may benefit from examining. Differences in inter-regional phase coupling between DS and TD groups also remains an important avenue for future investigation; however, there is a risk spurious increases in phase coupling may arise due to significant differences in oscillatory power between groups [40]. Such studies will therefore need to control for this.

Interestingly, we report here significantly lower alpha peak amplitude in individuals with DS. In line with this, previous research indicates that within individuals with DS, higher peak amplitude is associated with greater cognitive ability [19]. However, we also find here that theta power is significantly higher in individuals with DS compared to TD controls, despite previous research suggesting greater theta power may be associated with greater cognitive ability in individuals with DS [19]. It may therefore be the case that some EEG measures associated with higher cognitive ability in people with DS are closer to that of the TD population, whilst others are of the opposite direction (potentially suggestive of compensatory mechanisms). Importantly, therefore, higher ability individuals with DS may not necessarily have EEG activity closer to TD EEG spectra. The implications of this are interventions aiming to enhance cognitive ability in this population through seeking to ‘normalise’ EEG spectra could in fact negatively impact cognition. Instead, targeting EEG measures associated with individual differences in cognitive ability, rather than measures that differ between individuals with DS and TD controls, may be of benefit.

## Conclusions

We report an overall ‘slower’ EEG spectrum, characterised by higher delta and theta power, and lower alpha and beta power, for frontal and occipital regions in people with DS. Alpha activity in particular shows strong group differences, including lower power, lower peak amplitude and greater peak frequency variance in people with DS. Such ‘slowing’ of the EEG spectrum has previously been associated with cognitive decline in both DS and TD populations. These findings indicate the potential existence of a universal EEG signature of cognitive impairment, regardless of origin (neurodevelopmental or neurodegenerative), warranting further exploration.

## Supplementary Information

**Additional file 1.** Alpha power scalp plots. Scalp plots of alpha power contrasts for raw power group differences (left) and normalised power group differences (right).

## Abbreviations

AD: Alzheimer’s disease
ANCOVA: Analysis of covariance
CAMDEX-DS: Cambridge Examination of Mental Disorders of Older People with Down Syndrome and Others with Intellectual Disabilities
DS: Down syndrome
EEG: Electroencephalography
EC: Eyes closed
ID: Intellectual disability
MIPDB: Multimodal Resource for Studying Information Processing in the Developing Brain
SD: Standard deviation
TD: Typically developing
