# Supplementary figures and images for: Comparison of resting-state EEG between adults with Down syndrome and typically developing controls
Source: J Neurodev Disord. 2021 Oct 14;13:48. doi: 10.1186/s11689-021-09392-z (PMC8518326; doi:10.1186/s11689-021-09392-z)

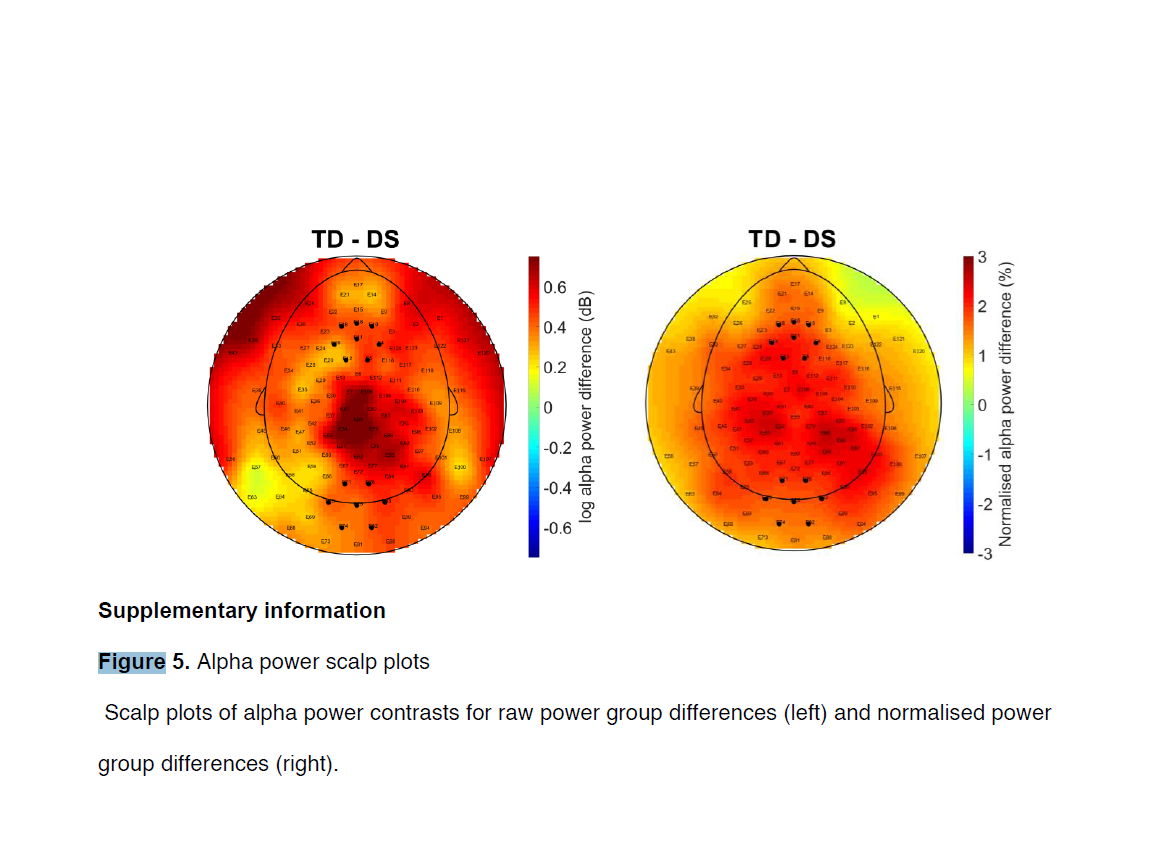

Supplement: Supplementary file 1 — Additional file 1. Alpha power scalp plots. Scalp plots of alpha power contrasts for raw power group differences (left) and normalised power group differences (right). [file 11689_2021_9392_MOESM1_ESM.png]
